# The Modulatory Effects and Therapeutic Potential of Cannabidiol in the Gut

**DOI:** 10.3390/cells13191618

**Published:** 2024-09-26

**Authors:** Kevin Brown, Kyle Funk, Alexa Figueroa Barrientos, Ashly Bailey, Sarah Shrader, Wenke Feng, Craig J. McClain, Zhao-Hui Song

**Affiliations:** 1 College of Medicine and Life Sciences, The University of Toledo, Toledo, OH 43614, USA; 2 Department of Pharmacology and Toxicology, University of Louisville School of Medicine, Louisville, KY 40292, USA; 3 Department of Structural and Cellular Biology, Tulane University School of Medicine, New Orleans, LA 70112, USA; 4 Division of Gastroenterology, Hepatology and Nutrition, Department of Medicine, University of Louisville School of Medicine, Louisville, KY 40202, USA

**Keywords:** cannabidiol (CBD), endocannabinoid system, Δ^9^-tetrahydrocannabinol (Δ^9^-THC), caco-2 cell, inflammatory bowel disease (IBD), Crohn’s disease, ulcerative colitis, gastrointestinal tract, microbiome

## Abstract

Cannabidiol (CBD) is a major non-psychotropic phytocannabinoid that exists in the *Cannabis sativa* plant. CBD has been found to act on various receptors, including both cannabinoid and non-cannabinoid receptors. In addition, CBD has antioxidant effects that are independent of receptors. CBD has demonstrated modulatory effects at different organ systems, such as the central nervous system, immune system, and the gastrointestinal system. Due to its broad effects within the body and its safety profile, CBD has become a topic of therapeutic interest. This literature review summarizes previous research findings with regard to the effect of CBD on the gastrointestinal (GI) system, including its effects at the molecular, cellular, organ, and whole-body levels. Both pre-clinical animal studies and human clinical trials are reviewed. The results of the studies included in this literature review suggest that CBD has significant impact on intestinal permeability, the microbiome, immune cells and cytokines. As a result, CBD has been shown to have therapeutic potential for GI disorders such as inflammatory bowel disease (IBD). Furthermore, through interactions with the gut, CBD may also be helpful in the treatment of disorders outside the GI system, such as non-alcoholic liver disease, postmenopausal disorders, epilepsy, and multiple sclerosis. In the future, more mechanistic studies are warranted to elucidate the detailed mechanisms of action of CBD in the gut. In addition, more well-designed clinical trials are needed to explore the full therapeutic potential of CBD on and through the gut.

## 1. Introduction to CBD and the Endocannabinoid System

*Cannabis sativa* has been used for medicinal and recreational purposes throughout the world for many centuries. The Cannabis plant is made up partially of phytocannabinoids. Two well-known phytocannabinoids that exist within the Cannabis plant are Δ^9^-tetrahydrocannabinol (Δ^9^-THC), the main psychoactive component of Cannabis, and cannabidiol (CBD), the main non-psychotropic phytocannabinoid [1]. CBD has been a topic of interest with regard to therapeutic uses and was recently approved by the Food and Drug Administration for the treatment of seizures involved in Lennox–Gastaut syndrome, Dravet syndrome, and tuberous sclerosis complex [2]. In this article, we first introduce the interaction of CBD with its molecular targets, followed by a brief introduction of the intestinal barrier and gut microbiome. We then focus on reviewing the results from preclinical and clinical research of the effects of CBD on intestinal permeability, gut microbiome, and potential therapeutic effects. Finally, we conclude by pointing out future directions for studies of CBD on the gut and its significance for health and the treatment of diseases.

The endocannabinoid system is a lipid signaling system that is composed of endocannabinoid ligands such as anandamide and 2-arachidonoylglycerol (2-AG), cannabinoid receptors such as CB1 and CB2, and enzymes that are responsible for the synthesis and degradation of endocannabinoids [3].

The endocannabinoid system is widely distributed in the body, and has been shown to affect an array of cognitive processes such as memory, reward, learning and to play an important role in the development of psychiatric disorders such as anxiety, depression, and schizophrenia [4]. In addition, the endocannabinoid system is involved in many other physiological processes and has been implicated in the development of a variety of pathological conditions such as neurological disorders and cardiovascular and gastrointestinal diseases [5,6].

## 2. Introduction to CBD Targets

### 2.1. CB1 and CB2 Cannabinoid Receptors

There are two well-characterized cannabinoid receptors, CB1 and CB2 [7,8]. Both CB1 and CB2 are G protein-coupled receptors and share 48% identity in amino acid sequences. However, they differ in terms of the locations in which they are expressed. CB1 receptors are in the brain, as well as in the peripheral nervous system and peripheral organs. CB2 receptors have been identified mainly in the tissues of the immune system [7,8]. CB2 receptors have also been shown to be expressed in the brain at a much lower level and have been thought to play an important role in counteracting neuroinflammation [9,10]. Regarding their signaling mechanisms, both CB1 and CB2 have been shown to act mainly through coupling to G_i/o_ proteins, and therefore, among others, inhibiting adenylate cyclase [7,8]. CBD has been shown to have low affinity toward the CB1 and CB2 receptors. Also, CBD has been demonstrated to exhibit negative allosteric or antagonistic effects toward both CB1 and CB2 [11,12,13].

In the gut, CB1 receptors are expressed in the enteric nervous system while CB2 receptors are primarily expressed in immune cells and to a lesser extent in the enteric nervous system [14].

### 2.2. Non-CB1/CB2 G Protein-Coupled Receptors

#### 2.2.1. GPR55

GPR55 is an orphan rhodopsin-like G protein-coupled receptor cloned by Sawzdargo et al. in the late 1990s [15]. Tissue distribution analysis showed GPR55 expression in various regions of the brain, such as the caudate and putamen, as well as in peripheral organs such as the spleen and intestines [15]. Later, GPR55 was suggested to be a novel, non-CB1/CB2 cannabinoid receptor [16,17]. It has been shown that CBD acts as an antagonist on GPR55 and recently, the antagonistic activity of CBD at GPR55 has been suggested as one of the mechanisms for the therapeutic effects of CBD in the treatment of epilepsy, specifically tuberous sclerosis, Dravet syndrome, and Lennox–Gastaut syndrome [18].

GPR55 is expressed in the gut and is thought to be involved in gastrointestinal functions including gut motility and intestinal inflammation [19]. Furthermore, GPR55 is expressed in specific regions of the brain responsible for appetite and satiety, suggesting that the receptor may also play a role in central regulation of food intake [19].

#### 2.2.2. GPR3, GPR6, and GPR12

GPR3, GPR6, and GPR12 are a group of orphan G protein-coupled receptors that are related to CB1 and CB2 cannabinoid receptors [20,21]. GPR3, GPR6, and GPR12 have all been shown to constitutively activate G_s_ proteins. In recent years, CBD has been found to be an inverse agonist for GPR3, GPR6, and GPR12 [20,22,23]. GPR3, GPR6, and GPR12 are expressed in various areas of the brain and peripheral tissues, where they play important roles in pathological conditions such as Alzheimer’s disease, Parkinson’s disease, addiction, obesity, and cancer [20,21]. The discovery that GPR3, GPR6, and GPR12 are molecular targets for CBD provided important mechanistic insight for understanding the therapeutic potential of CBD for various diseases, including Alzheimer’s disease and Parkinson’s disease [20,22,23]. To date, there has been no report describing the expression of these receptors in the gut.

#### 2.2.3. 5-HT1a Receptor

The 5-HT1a receptor is a G_i/o_- coupled receptor that functions to inhibit adenylate cyclase. 5-HT1a receptors are expressed as both pre-synaptic and post-synaptic receptors within the brain, making them relevant in treating psychiatric disorders [24]. CBD was found to have moderate affinity and display agonist activity at 5-HT1a receptors [25]. The agonistic activity of CBD at 5-HT1a has been suggested as the basis behind the anxiolytic effect of CBD [26].

5-HT1a receptors are widely distributed in the enteric nervous system that controls gastrointestinal functions and are involved in regulating gut motility and secretion [27]. In addition, these receptors contribute to visceral sensitivity, affecting how the gut perceives pain and discomfort. Drugs targeting 5-HT1a receptors are used in a variety of conditions, including irritable bowel syndrome (IBS) [28,29].

#### 2.2.4. Opioid Receptors

Opioid receptors belong to the G protein-coupled superfamily of receptors and include the mu, kappa, and delta receptors [30,31]. Kathmann et al. found that CBD binds allosterically to mu and delta opioid receptors and modulates ligand binding activity at these receptors [32]. A later study found that CBD treatment reduced ethanol consumption and relapse in mice, and CBD also significantly reduced expression of several receptor genes, including mu-opioid receptors [33].

Opioid receptors are present in the enteric nervous system as well as the central nervous system. Opioids can modulate the gut–brain axis by producing dysbiotic effects and inducing an increase in intestinal permeability. Components of bacteria can modify the expression of opioid receptors and cause increased sensitivity to pain [34,35]. Opioids are well known to cause constipation by activating µ-opioid receptors in the gut, which lengthens the transit time of gut contents [34,35]. Therefore, targeting specific opioid receptor subtypes may help manage conditions like chronic constipation, IBS, and inflammatory bowel disease [34,35].

### 2.3. Other CBD Targets

#### 2.3.1. PPAR-γ

The PPAR-γ receptor is a ligand-activated transcription factor expressed in a variety of tissues, including adipose tissue and the central nervous system [36]. In the adipose tissue, PPAR-γ assists in increasing insulin sensitivity and in the metabolism of lipids [37]. In the central nervous system, PPAR-γ plays a number of important roles, including attenuating the neuroinflammatory response [38]. CBD has been demonstrated to display agonist activity at PPAR-γ [36]. Through its interaction with the PPAR-γ receptor, CBD has been found to promote lipogenesis in mouse and human mesenchymal stem cells [37]. In the rat brain, CBD protects against neuroinflammation and neurotoxicity induced by β-amyloid, and this protective effect of CBD was inhibited by a PPAR-γ antagonist [39].

PPARγ is highly expressed in the gut, particularly in epithelial cells and immune cells, and plays a crucial role in regulating intestinal inflammation [40]. Activation of PPARγ alleviates nuclear factor-κB (NF-κB)-mediated inflammation. Therefore, both natural and synthetic PPARγ ligands are potential therapeutic agents for the treatment of colitis [41,42].

#### 2.3.2. TRPV

The transient receptor potential vanilloid (TRPV) delineates one of the seven subfamilies of receptors within the transient receptor potential (TRP) family [43]. TRPV1, TRPV2, TRPV3, and TRPV4 are expressed in trigeminal and dorsal root ganglia, as well as in the brain. These receptors have been demonstrated to play a role in the sensation of heat and pain. In addition, TRPV3 is expressed in the skin and hair, and contributes to healing of wounds and the growth of hair. Also, TRPV4 is expressed in the bones, playing an important role in bone development and health [43]. CBD has been found to display agonistic activity on TRPV 1–4 [44,45,46]. For example, the interaction of CBD with TRPV1 can lead to pain relief.

TPRV1 and TPRV4 are expressed in the intestines and are potential therapeutic targets for inflammatory bowel disease [47,48]. It has been shown that both TRPV1 and TRPV4 exert pro-inflammatory effects in the intestines. Thus, antagonists of these two channels are anti-inflammatory [49,50].

#### 2.3.3. CBD as an Antioxidant

The antioxidant properties of CBD are attributed to both its molecular structure and its ability to induce the expression of endogenous antioxidant proteins. First, CBD can participate in antioxidant reactions through its aromatic electrophilic center and hydroxyl nucleophilic properties [51,52]. This enables CBD to scavenge for ionic metals and chelate them before they enter the Fenton reaction, thus decreasing the production of reactive oxygen species (ROS). Secondly, CBD also works to increase gene expression of enzymes in the antioxidant systems such as superoxide dismutase and glutathione peroxidase in several tissues [51,52]. However, the impact of CBD on the gene expression of these two enzymes specifically in the gut remains unknown.

This section described the molecular targets of CBD and their expression in the GI system. Some of these molecular targets of CBD have been shown to be involved in the actions of CBD in a model of intestinal epithelial cells (described in Section 5 of this review). However, currently, the in vivo involvement of these molecular targets in the actions of CBD in the GI tract is not clear and remains to be studied using approaches such as gene knockout of these molecular targets.

## 3. CBD Pharmacokinetics

CBD can be administered via different routes including oral, inhalation, intravenous, and transdermal [53]. The degree of absorption is determined by the routes of administration, with oral administration leading to the lowest plasma concentration, and the intravenous injections reaching the highest plasma concentration. CBD is highly lipophilic, thus multiple formulations for oral administration have been investigated to increase its bioavailability, such as gelatin matrix pellets, lipid/oil-based formulations, and self-emulsifying drug delivery systems. Impaired liver function, female sex, and food/fat intake have demonstrated to increase absorption of CBD via the oral route. Administration methods such as inhalation, transdermal, and intravenous avoid first-pass metabolism of the oral routes, increasing CBD plasma concentration [53].

CBD interacts with several enzymes and transporters that are important for pharmacokinetics. These include the cytochrome P450 (CYP) family, carboxyl esterase 1, glucuronyl transferases, and P-glycoprotein [54]. CBD is metabolized in the liver by the CYP450 enzyme family, more specifically CYP2C19 and CYP3A4, along with other isoenzymes. Specifically, CYP2C19 hydroxylates CBD at the 7 position, transforming it to its active metabolite 7-OH-CBD [55]. Drug-to-drug interaction has shown altered CBD metabolism, for example, CYP enzyme inducers such as Rifampicin yield lower CBD plasma concentration, whereas CYP enzyme inhibitors such as Ketoconazole result in higher levels of CBD present in plasma [56]. Thus, dose adjustment should be considered when administering CBD together with other drugs that share metabolism with CYP450 enzymes. Most CBD and is metabolites are excreted via feces with minimal urinary excretion [53].

## 4. Introduction to the Intestinal Barrier and Gut Microbiome

The human gut encounters many potentially toxic, detrimental substances on a regular basis and requires an effective way of maintaining homeostasis when exposed to these agents. The barrier within the intestine is composed of a superficial apical layer and a deep basolateral layer, which together serve an important role in inhibiting the entrance of harmful substances into the bloodstream [57,58,59,60]. Conversely, the intestinal layers also interact and communicate in a dynamic manner to allow beneficial nutrients to pass through the barrier and reach the circulation. The ability of the intestinal barrier to differentiate between beneficial and harmful substances is critical to maintaining homeostasis and preventing disease processes [57,58,59].

Each specific component of the intestinal barrier, which consists of the bacterial microbiota, mucus layer, intestinal epithelial cells, and lamina propria, plays a specific role in maintaining intestinal tract health [61,62,63,64,65,66]. There are several cell types found in the intestinal epithelial cell layer, which include goblet cells, Paneth cells, enterocytes, neuroendocrine cells, and stem cells. Goblet cells secrete mucus and antimicrobial proteins; Paneth cells produce antimicrobial proteins and peptides; neuroendocrine cells secrete peptides and hormones that exhibit protective functions; and enterocytes (simple columnar epithelial cells) play important roles in adsorption of nutrients and secrete chloride and water when encountering potentially harmful substances [61,62,63,64,65,66,67]. The permeability between the intestinal epithelial cells is closely regulated by the tight junctions, adherens junctions, desmosomes, and gap junctions that connect these cells to one another [61]. Dysregulated permeability of the intestinal barrier has been shown to be associated with disorders of the intestinal tract, including inflammatory bowel disease (IBD) [61]. Interestingly, this increased permeability has also been linked with other diseases such as obesity, diabetes mellitus, and heart disease [57,58,59].

The gut microbiome plays a crucial role in the overall homeostasis of the intestinal barrier. The various microbes within the gut play important functions, such as maintaining appropriate intestinal permeability, regulating gene transcription and translation, modulating the immune system, and producing anti-inflammatory effects [68].

## 5. Introduction to Inflammatory Bowel Disease

Inflammatory bowel disease (IBD) is a term used to describe a chronic inflammatory disease process that affects the gastrointestinal tract and includes both ulcerative colitis and Crohn’s disease [69,70,71]. These two diseases can be distinguished from one another by unique features involving the location of the pathology and lesion depth in the gastrointestinal tract. Ulcerative colitis most often begins in the rectum, typically progressing upward through part of or the entire colon, and is defined by widespread inflammation solely of the mucosal layer. Crohn’s disease is characterized by inflammation involving all layers of the bowel wall and can affect any part of the gastrointestinal tract. The most commonly affected areas in Crohn’s disease are the colon and distal portion of the ileum [72]. The specific pathogenesis of IBD is not known, but is believed to include a wide combination of factors involving the immune system, genetics, microorganisms, and environmental factors. IBD is usually characterized by periods of relapsing and remitting disease activity, and the disease can have a tremendously negative impact on an individual’s quality of life [73].

A curative treatment does not currently exist for IBD, and treatment is centered around producing a sustained remission from symptoms [74]. There are several classes of medications that are employed for treatment, which include corticosteroids, 5-aminosalicylic acid drugs, oral small molecule drugs, immunomodulators, and biologic agents [75]. However, many of these drugs are associated with significant side-effects. Previous research has found that the use of cannabis for symptom relief is relatively common in patients with IBD, especially in those with more severe symptoms and increased morbidity [76,77].

## 6. The Role of CBD in Inflammatory Bowel Disease

Recently, CBD has been a topic of high interest for its possible role in the treatment of IBD. The following sections of this article explore pre-clinical and clinical findings regarding the efficacy of CBD in treating IBD. This section describes previous research that has investigated the effects of CBD in the treatment of gastrointestinal inflammatory diseases, including studies using cell culture and animal models, along with human clinical trials.

### 6.1. The Effect of CBD on Intestinal Epithelial Cells

All the in vitro studies included in this review article utilized the caco-2 cell line. The caco-2 cell line was derived from a carcinoma of the colon in the 1970s [78]. This cell line possesses the ability to differentiate into a monolayer of cells with a polarized columnar epithelium, as is found in the human small intestine [78]. Due to their similarities to intestinal epithelial cells, differentiated caco-2 cells are widely used as a model of intestinal permeability and in the evaluation of drug absorption [79].

#### 6.1.1. Effects of CBD in Regulating Intestinal Permeability

**Figure 1:**
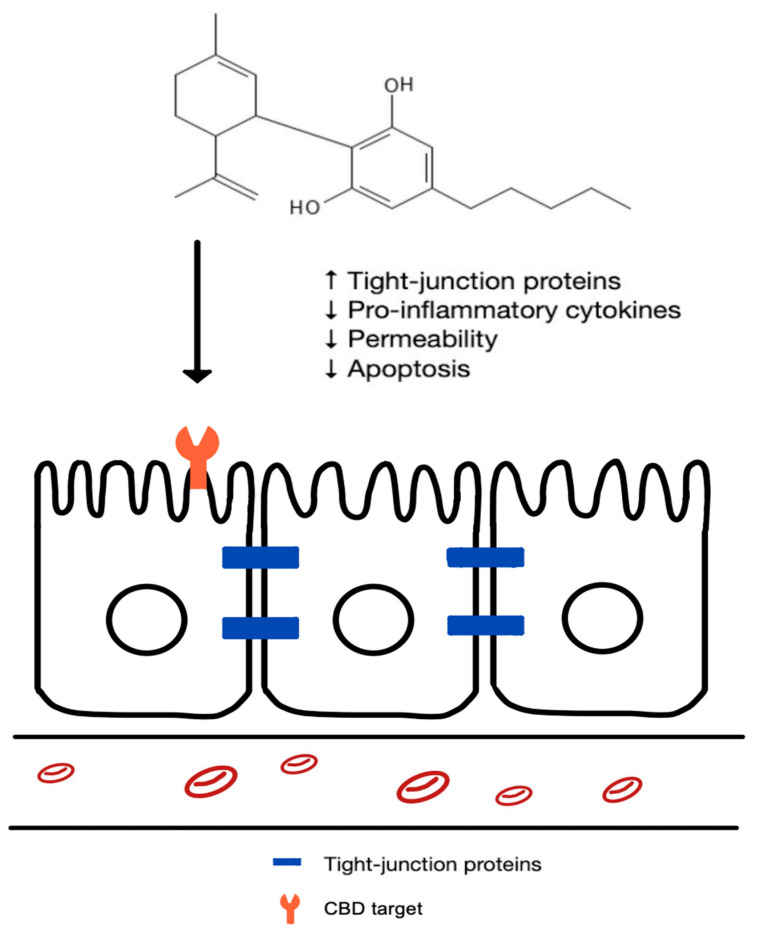
The effects of CBD on intestinal epithelial cells.

Over the years, O’Sullivan’s group published a series of reports on the effects of CBD on cultured caco-2 cells (Figure 1 and Table 1). In general, CBD was shown to decrease membrane permeability as demonstrated by the increase in transepithelial electrical resistance (TEER) and the upregulation of tight-junction proteins. For example, in a study published in 2010, EDTA was used to induce an increase in caco-2 cell permeability as indicated by a reduction in TEER [80]. By interacting with CB1 and TRPV1, CBD resulted in an upregulation in zona occludens-1 expression, and a decrease in permeability as indicated by an increase in TEER. In 2012, combined use of cytokines TNF-α and INF-γ was reported in a paper published by O’Sullivan’s group (and many other papers from this group) to model inflammation [81]. It was concluded that via CB1 receptor interaction, CBD decreased the inflammation-induced enhancement in permeability, as evidenced by a CBD-induced increase in TEER. Later, in 2019, O’Sullivan’s group reported that CBD activated adenylate cyclase through CB1 receptors. Moreover, CBD prevented the decrease in mRNA for claudin-5. Again, it was demonstrated that CBD decreased permeability, as shown by the decreased flux of dextran [82].

Other groups have published data that are consistent with the results reported by O’Sullivan’s group on the modulatory effects of CBD on caco-2 cell monolayer permeability. For example, Cocetta et al. (2021) found that CBD prevented a decrease in TEER caused by pro-inflammatory cytokines [83]. Also, a study conducted by Gigli et al. (2017) showed that CBD treatment induced an increase in TEER and tight-junction proteins in caco-2 cells infected with *Clostridioides difficile* (formerly known as *Clostridium difficile*) toxin, and the effects of CBD were blocked by a CB1 antagonist [84]. Finally, Corpetti et al. (2021) demonstrated that CBD increased the expression of tight-junction proteins, along with the repair of the TEER and fluorescein isothiocyanate-dextran permeability of caco-2 cells incubated with SARS-CoV-2 spike protein. These specific effects were mediated by CBD interactions with PPAR-γ [85]. From a clinical perspective, these studies collectively suggest that CBD may have therapeutic potential in the management of inflammatory states that disrupt intestinal epithelia cell permeability.

**Table 1:** Studies concerning the effects of CBD on differentiated caco-2 cells.

Stimuli	CBD Effect	Receptors Involved	Therapeutic Relevance	References
50 μM EDTA	↑ zona occludens-1 expression ↓ permeability with an associated ↑ in TEER	CBD interacts with CB1 on the apical membrane and TRPV1 on the basolateral membrane of caco-2 cells. Application of receptor antagonists on their respective sides prevented the effect of CBD.	CBD may be an effective agent for the treatment of IBD by altering intestinal permeability	[80]
10 ng/mL TNF-α and 10 ng/mL INF-γ	↓ permeability with an associated ↑ in TEER	CBD interacts with CB1. Application of a CB1 antagonist prevented the effect of CBD.	CBD may be an effective agent for the treatment of IBD by reducing cytokine-induced increase in intestinal permeability	[81]
10 ng/mL TNF-α and 10 ng/mL INF-γ	↓pro-inflammatory protein phosphorylation ↓ production of cytokines.	CBD interacts with CB2 and TRPV1. Application of CB2 and TRPV1 antagonists prevented the effect of CBD.	CBD may be an effective agent for the treatment of IBD through anti-inflammation	[86]
10 ng/mL TNF-α and 10 ng/mL INF-γ	Prevented decreases in claudin-5 mRNA, ↓ the flux of dextran, ↓ absorption of lactulose and mannitol	CBD interacts with CB1 to activate adenylyl cyclase activity. The effect of CBD was inhibited by CB1 antagonism.	CBD may be an effective agent for the treatment of IBD by reducing cytokine-induced increase in intestinal permeability	[82]
10 ng/mL INF-γ and 10 ng/mL TNF-α	Inhibition of ROS production, ↑ in TEER, modulation of markers of intestinal dysfunctions, and restoration of epithelial permeability during times of high inflammation or oxidative stress	Not studied.	CBD may be an effective agent for the treatment of IBD by reducing cytokine-induced oxidative stress and increase in intestinal permeability	[83]
30 ng/mL *Clostridioides difficile* toxin A	↓ apoptotic process induced by *Clostridioides difficile* toxin A. ↑ viability of caco-2 cells, restored TEER, and ↑ the expression of RhoA-GTPase and tight-junction proteins	CBD interacts with CB1. Application of a CB1 receptor antagonist prevented the effect of CBD.	CBD may be an effective agent for the treatment of GI diseases induced by *Clostridioides difficile*	[84]
Spike protein from SARS-CoV-2	↓ in all pro-inflammatory markers ↑ expression of tight-junction proteins and restoration of TEER ↓ TLR-4 and angiotensin converting enzyme 2 (ACE-2).	CBD interacts with PPAR-γ. The effect of CBD was prevented by a PPAR-γ antagonist.	CBD may have therapeutic potential for treating enterotoxicity induced by SARS-CoV-2 spike protein	[85]

Symbols and abbreviations used: ↑ increase; ↓ decrease; TEER, transepithelial electrical resistance; TRPV1, transient receptor potential vanilloid subtype 1; TLR-4, toll-like receptor 4; PPAR-γ, peroxisome proliferator-activated receptor gamma; GTP, guanosine triphosphate; GI, gastrointestinal; RhoA-GTPase, Ras homologues A-GTPase; IBD, inflammatory bowel disease; TNF-α, tumor necrosis factor alpha; INF-γ, interferon gamma; ROS, reactive oxygen species.

#### 6.1.2. Effects of CBD on Intestinal Pro-Inflammatory Cytokine and Cell Apoptosis

Several studies have demonstrated the beneficial effects of CBD in reducing intestinal inflammation and apoptosis. For example, in 2017, O’Sullivan’s group reported the inhibitory effects of CBD on phosphorylation of pro-inflammatory proteins and production of cytokines in differentiated caco-2 cells [86]. In addition, it was determined that CBD acts on CB2 and TRPV1 receptors to produce its effects.

The study by Gigli et al. in 2017 showed that toxin A from the bacterium *Clostridioides difficile* increased the frequency of apoptosis [84]. By interacting with CB1 receptors, CBD effectively counteracted detrimental effects by *Clostridioides difficile* toxin A, showing an increase in the viability of caco-2 cells and a decrease in the expression of Bax protein, which functions in apoptotic processes. Additionally, toxin A-infected caco-2 cells treated with CBD resulted in higher expression levels of RhoA GTP, a regulator protein essential for proliferation and homeostasis in the intestines. Overall, this study demonstrated that CBD could have therapeutic potential for decreasing *Clostridioides difficile* toxin A-induced intestinal cell death.

A study published in 2021 showed that CBD interactions with PPAR-γ diminished all the pro-inflammatory markers that were stimulated by incubation of the SARS-CoV-2 spike protein with caco-2 cells [85]. This study suggests that CBD has therapeutic potential for the intestinal inflammation caused by the SARS-CoV-2 spike protein.

Altogether, these caco-2 cell culture studies suggest the therapeutic potential for CBD in treating IBD and toxin-induced intestinal disorders characterized by inflammation and changes in intestinal permeability (Figure 1 and Table 1). More specifically, CBD can increase certain tight-junction proteins, decrease intestinal permeability, and increase TEER values. In addition, CBD can prevent cell apoptosis, decrease reactive oxygen species, and inhibit pro-inflammatory cytokines. Regarding mechanisms of action, these studies demonstrate that CBD interacts with a variety of receptors, including CB1, CB2, TRPV1, and PPAR-γ, to exert its effects on intestinal epithelial cells.

### 6.2. Animal Studies Regarding the Therapeutic Potential of CBD for IBD

Over the years, several groups have conducted pre-clinical studies investigating the potential effects of CBD on inflammatory diseases of the gastrointestinal tract (Table 2). By inducing colitis in mice, these studies were able to measure various metrics related to the reduction in inflammation following treatment with CBD. The interesting data collected in these studies are highly useful in terms of setting a base for the future clinical research exploring the effectiveness of CBD on the management of gastrointestinal inflammation.

Borrelli et al. (2009) conducted a study using a dinitrobenzene sulfonic acid (DNBS)-induced IBD model in mice [87]. Mice treated with DNBS showed an increase in colon weight/length ratio, which was effectively reduced by CBD. Also, treatment with CBD led to reduced swelling and increased gland regeneration in the intestine, indicating a reversal in colonic injury. In addition, CBD significantly reduced the expression of iNOS, which was overexpressed in this IBD model, and reverted the abnormal cytokine levels (elevated IL-1β and reduced IL-10) in the colonic tissue closer to baseline levels. This study demonstrated that CBD is effective in treating IBD in this pre-clinical model [87].

Using lipopolysaccharide (LPS)-treated mice, De Filippis et al. (2011) investigated the effects of CBD on enteric glial cell (EGC) activation [88]. LPS-treated mice experienced an increase in expression of the glial cell marker S100B, which is exclusively produced in glial cells of the intestine. When mice were treated conjunctively with LPS and CBD, there was a significant reduction in S100B expression, indicating a reduction in glial proliferation. Immune cells, such as mast cells and macrophages, became activated in LPS-treated mice. With the addition of CBD, both the activation and prevalence of these cells were significantly reduced. Caspase 3 is a protein that promotes apoptosis when cleaved. In this study, LPS-treated mice were shown to have increased expression of cleaved caspase-3 proteins and these levels were significantly reduced by CBD. Overall, it was concluded that CBD has the potential to act as a physiological antagonist to EGC activation and immune response, and to reduce LPS-induced damage to the intestine [88].

In contrast to the two studies described above, a study by Becker et al. (2021) using clinical parameters of colitis assessed by colonoscopy, histology, flow cytometry, and serum biomarkers, found a lack of therapeutic efficacy for CBD in both 2,4,6-trinitrobenzenesulphonic acid (TNBS)-induced and dextran sulfate sodium (DSS)-induced colitis mouse models [89]. This study showed that Δ^9^-THC prevented colitis by acting through CB2 receptors to alter both immune cell and enterocyte activity. CBD, on the other hand, was not effective in preventing colitis either alone or when combined with Δ^9^-THC [89]. Currently, it is unknown why CBD was not effective in this study. The efficacy of CBD may be dependent on the model or the delivery method used.

A study was performed by Silvestri et al. (2020) using a dextran sulfate sodium (DSS)-induced colitis model in mice to study the ability of fish oil and CBD to reduce inflammation [90]. It was found that CBD alone did not reduce colitis severity. However, when a per se ineffective dose of 20 mg of fish oil was co-administered with CBD, significant reductions in disease activity index, colon weight/length ratio, and myeloperoxidase activity were observed. Combined treatment with fish oil and CBD also led to significant reductions in the elevated inflammatory markers in this colitis model. Notably, the fish oil treatment alone was ineffective at 20 mg. This study provided evidence for the therapeutic potential of co-administration of fish oil and CBD in the treatment of IBD [90].

Overall, these pre-clinical investigations suggest that CBD can produce beneficial effects in managing IBD (Table 2). CBD had the capability to reduce colonic weight/length ratio and the intestinal permeability. In addition, CBD was able to reduce inflammatory cytokines, iNOS, and immune cells, such as macrophages and mast cells, within the intestines. However, some of these studies found that CBD was not effective in IBD models, or CBD was effective only when combined with fish oil. These previous studies used different doses and routes of administration of CBD, which may contribute to the variations in results. Further studies are needed to clarify the effectiveness of CBD for IBD.

### 6.3. Human Clinical Studies Investigating the Effect of CBD on IBD

In this section, we summarize the two clinical trials that have studied the effects of CBD on IBD (Table 3).

A randomized, placebo-controlled trial was conducted by Naftali et al. on 19 patients to investigate the efficacy of CBD in treating Crohn’s disease [91]. Treatment with 10 mg of CBD oil twice daily was well tolerated, with no significant alterations in liver or kidney functions or blood cell counts. However, there were also no significant differences in the Crohn’s disease activity index between the CBD oil and the placebo group. It was concluded that the low-dose CBD treatment was not effective in the treatment of Crohn’s disease. Additional studies using higher doses of CBD are needed to form stronger conclusions on the usefulness of CBD in the treatment of IBD. Further studies using novel delivery methods may improve the efficacy of CBD.

Irving et al. performed a randomized study on the effects of CBD-rich extract on 39 patients with mild to moderate ulcerative colitis [92]. It was found that 77% of participants in the CBD group had disease scores in the normal and mild range, compared to 52% in the control group. Participants in the CBD group showed higher quality of life scores compared to those in the control group. In addition, 66% of CBD-treated patients had improved endoscopic subscores by the end of the trial, compared to 38% in the placebo group. Furthermore, circulating concentrations of inflammatory cytokines such as IL-6, IL-2, and TNF-α were reduced in the CBD-treated group. Overall, this study demonstrated that for patients with ulcerative colitis, CBD has the potential to reduce severe symptoms such as hemorrhagic diarrhea and to improve patients’ quality of life.

In conclusion, two clinical studies on the effects of CBD in the treatment of IBD displayed varying results (Table 3). For Crohn’s disease, CBD was not effective. For ulcerative colitis, CBD-rich cannabis extract was effective. Given the debilitating impact of IBD, the potential efficacy of CBD for IBD treatment holds a high level of clinical relevance. Further clinical trials are warranted to study the effects of CBD for IBD with larger patient populations, varying doses, and different formulations of CBD.

## 7. The Effect of CBD on the Gut Microbiome

### 7.1. Introduction to the Gut Microbiome

The makeup of an individual’s gut microbiome is influenced by both external environmental factors and genetics. One of the major external factors that has been shown to be a strong influence on the microbiome is diet [93]. A balanced, healthy diet leads to microbiome symbiosis through the production of metabolites such as short-chain fatty acids (SCFAs), indole compounds, and secondary bile acids. These metabolites play essential roles in maintaining gut homeostasis and effective regulation of the immune response. SCFAs, including butyrate, influence immune responses in a number of ways. For example, they promote regulatory T cell (Treg) differentiation, which helps suppress excessive inflammation. Also, butyrate inhibits pro-inflammatory cytokines (such as TNF-α and IL-6) and enhances anti-inflammatory cytokines (e.g., IL-10) [94,95]. Furthermore, butyrate is a key player in gut health, as it serves as the primary energy source for colonic epithelial cells. By promoting colonic epithelial cell proliferation and differentiation, and enhancing tight-junction integrity, butyrate helps maintain a healthy intestinal barrier, preventing unwanted substances (like pathogens or toxins) from crossing the epithelium. Butyrate also modulates mucus production, supporting the protective mucus layer [94,95].

An unbalanced and unhealthy diet, however, can cause dysbiosis, which can then lead to dysregulation of the intestinal barrier and immune system. This can result in the individual being vulnerable to chronic inflammation through an increase in virulence factors and an absence of immune regulatory factors [96,97]. For example, loss of butyrate-producing bacteria (e.g., *Roseburia*, *Faecalibacterium*) can lead to dysfunctional intestinal barrier (leaky gut) due to impaired tight junctions, and dysbiosis can cause chronic activation of pro-inflammatory pathways [94,95].

### 7.2. Studies Regarding the Effect of CBD on Intestinal Microbiome

**Figure 2:**
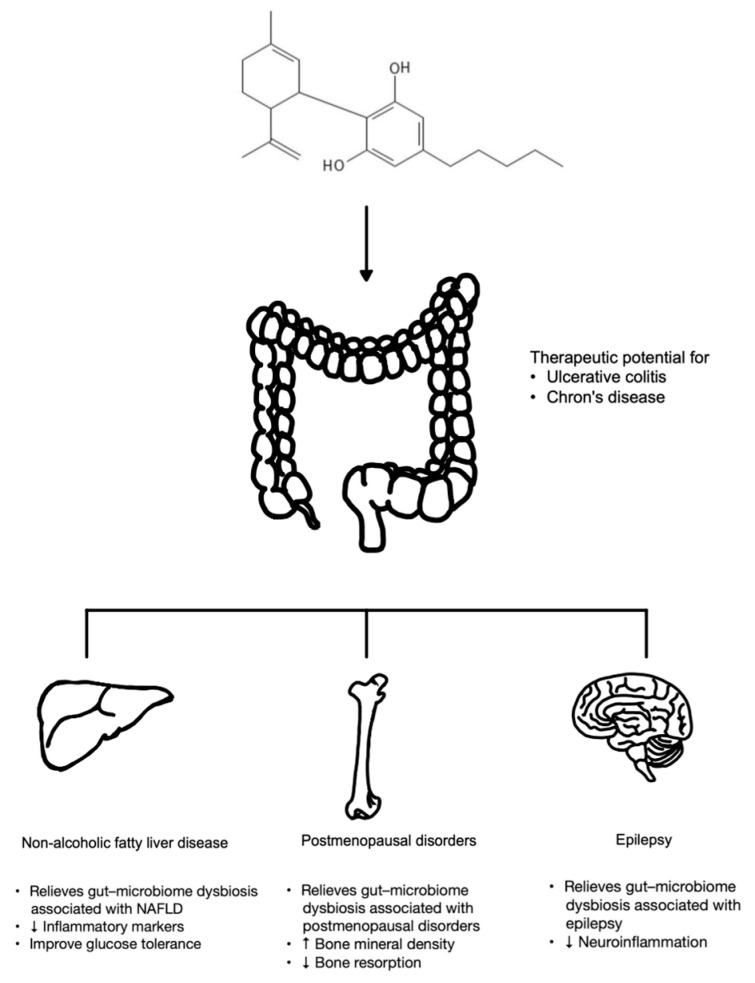
The potential therapeutic effects of CBD that are possibly mediated through its effects on gut microbiome.

This section describes previous studies investigating the effects of CBD on various specific elements of the gut microbiome. Since the microbiome has an impact on the health of multiple organ systems, these studies have also examined the correlation between the effects of CBD on the gut microbiome and its impact on diseases such as IBD, non-alcoholic fatty liver disease (NAFLD), postmenopausal disorders, epilepsy, and multiple sclerosis (Figure 2 and Table 4).

Silvestri et al. (2020) conducted a study using DSS-induced colitis in mice [90]. They found that even at per se ineffective doses for colitis, CBD alone had significant effects on the microbiome of the mice [90]. An increase in *Akkermansia muciniphilia*, a bacterial species associated with beneficial effects during gastrointestinal inflammation, was seen on day 8 of the colonic inflammation model following administration of CBD either alone or in combination with fish oil. The increase in *A. muciniphilia* on day 8 occurred prior to the inflammation resolution phase. Additionally, CBD administration, independently or in combination with fish oil, led to an increase in another bacterial species known for its beneficial effects during gastrointestinal inflammation, *Parabacteroides goldsteinii*, on day 8 of the study. Both *A. muciniphilia* and *Parabacteroides goldsteinii* are beneficial for gut health. They are anti-inflammatory and have protective effects on gut permeability [98,99,100].

Cumulatively, the findings suggest that CBD, administered either independently or with fish oil, may play a beneficial role in altering the gut microbiome during times of inflammation, and that future human studies on this subject are warranted [90].

The composition of the gut microbiota evolves throughout life, and the ratios of different bacteria change during different life stages. During infancy and old age, the *Firmicutes*/*Bacteroidetes* ratios are rather low, averaging about 0.4 and 0.61, respectively. However, during adulthood, the average ratio is 10.9. Dysbiosis in the *Firmicutes*/*Bacteroidetes* ratio can impact health, with increased ratios associated with obesity and decreased ratios linked to inflammatory bowel disease (IBD) [101,102]. Gorelick et al. (2022) conducted a study to evaluate the potential therapeutic effects of CBD on NAFLD progression [103]. NAFLD was induced in mice by feeding them with a high fat-cholesterol diet (HFCD) for 6 weeks. It was observed that the diet supplemented with CBD significantly reduced the abundance of *Deferribacteres*, which had been increased by the HFCD. In addition, CBD significantly increased abundance of *Firmicutes*, which had been decreased by HFCD. Overall, this led to an increased *Firmicutes/Bacteroidetes* ratio. CBD also increased the levels of *Clostridia*, *Ruminococcaceae*, and *Bilophila*, and inhibited the HFCD-mediated increase in the genus *Mucispirillum* and species *Mucispirillum schaedleri*. Importantly, this study demonstrated that CBD mitigated a number of measurements in the HFCD model, e.g., CBD improved glucose tolerance and reduced some of the inflammatory response makers such as tumor necrosis factor alpha (TNF-α) and iNOS. By showing that CBD ameliorated HFCD-associated alterations to the gut microbiome, this study suggests that CBD may play an effective role in alleviating the symptoms associated with NAFLD [103].

Sui et al. (2022) used a model of ovariectomized mice to investigate the possible therapeutic effects of CBD on the postmenopausal physiological changes [104]. The results of this study demonstrated that CBD altered the microbiome in the ovariectomized mice. Specifically, compared to the vehicle-treated group, the CBD-treated ovariectomized mice displayed a bloom of the *Lactobacillus* species within the intestines. Previous literature has shown that *Lactobacillus* has protective effects on bone health and prevents bone loss in models of ovariectomized mice [105,106]. Furthermore, a human study showed that the 75-to-80-year-old women who received supplementation with *Lactobacillus* had reduced loss of total bone density compared to the placebo group [107]. This study suggests that CBD may alter the gut–bone axis, which can subsequently improve postmenopausal bone health [104].

A study by Gong et al. (2022) investigated the effects of CBD on microbiome composition and inflammation of rats with epilepsy [108]. An epileptic rat model was generated by intraperitoneal injections of lithium chloride and pilocarpine. Four experimental groups were used: control, epileptic model, epileptic plus low-dose CBD, and epileptic plus high-dose CBD groups. It was found that the low- and high-dose CBD groups experienced seizures later and less severely compared to the epileptic model group that did not receive CBD treatment. Additionally, the low- and high-dose CBD groups had significantly lower levels of inflammatory factors, including IL-6, IL-β, and TNF-α, compared to those in the epilepsy model group. Regarding microbiome composition of the various groups, it was found that *Helicobacteraceae* abundance was significantly increased in the high-dose CBD group compared to the model group. Additionally, it was determined that epileptic rats treated with CBD showed a restoration in the abundance of the bacteria *Prevotellaceae UCG-100* compared to the model group. This bacterium has been shown to have beneficial immune effects in the host, including anti-inflammatory effects and increased defense against harmful pathogens. It is known that inflammation, which is associated with epilepsy, can have marked impacts on the composition of the microbiome. Since CBD has been shown to have anti-inflammatory effects, it potentially could be used therapeutically to reduce dysbiosis and remodel the gut microbiome in patients with epilepsy.

Dopkins et al. studied the effects of CBD on microbiome composition and inflammation in mice with experimental autoimmune encephalomyelitis (EAE), a model for multiple sclerosis (MS) [109]. To induce EAE in mice, animals were injected with myelin oligodendrocyte glycoprotein, Freund’s adjuvant, and dead *Mycobacterium tuberculosis*. CBD-treated mice showed decreased expression of CXCL9, IL-1β, and CXCL10 in infiltrating and resident myeloid cells of the nervous system compared to the control EAE model group. In addition, CBD-treated mice had lower expression of STAT proteins, most notably STAT1, compared to the control EAE model group. STAT1 is an antiviral protein that has been shown to increase inflammation, especially within the nervous system. Furthermore, anti-inflammatory effects were also found within the gastrointestinal system of the CBD-treated mice. Most notably, CBD was able to reduce the expression of gasdermins by inhibiting transcription of the gene in the intestine of the mice. In this study, although CBD showed promise in alleviating neuronal and GI inflammation in EAE mice, it seemed to have no effect on the microbiome composition. Using 16s rRNA sequencing, an amplicon sequencing method commonly used to study microbial diversity, the researchers found that there were no significant changes in cecal microbiota of CBD-treated mice compared to the control EAE model group. The researchers also studied beta diversity between the groups at different timepoints, but again there were no significant differences found. Additionally, microbiota metabolites were investigated by measuring the amount of short-chain fatty acids (SCFAs) within the cecum. Again, there were no significant differences in SCFA concentration between the CBD and control EAE model groups. Overall, it was concluded that CBD was effective in reducing neuroinflammation and GI inflammation in EAE, but it had little effect on the composition and metabolism of the gut microbiome [109].

The intestinal mucus layer consists mainly of high molecular weight glycoproteins (mucin), which provide physical protection by forming a gel-like layer that covers the gut epithelium. This layer acts as a barrier between the gut epithelial cells and the luminal contents, including gut microbes, toxins, and other irritants. When the mucus barrier is compromised, gut microbes can adhere to and invade the epithelium, leading to inflammation and disease. For example, conditions like Crohn’s disease and ulcerative colitis are associated with mucus barrier dysfunction [98,99,100].

*A. muciniphila* is an anaerobic bacterium that thrives on mucin. It degrades mucin into metabolites like short-chain fatty acids (SCFAs), including acetate and propionate. These SCFAs have anti-inflammatory and metabolic effects. *A. muciniphila*’s ability to produce SCFAs contributes to its beneficial impact on weight regulation, immune modulation, and improved intestinal barrier integrity. Thus, having more *A. muciniphila* can be advantageous. However, since *A. muciniphila* degrades mucin for fuel, too much *A. muciniphilia* could be detrimental to gut health [98,99,100].

A study by Skinner et al. administered CBD-rich cannabis extract to mice to test the effects of CBD on gut microbiome and health [110]. First, the study demonstrated that there was an increase in the abundance of *Akkermansia muciniphilia* following administration of CBD. Secondly, there was an increased abundance of multiple pro-inflammatory cytokines and chemokines. Thirdly, there was a reduced expression of the *Muc2* gene in the colon, which is a gene that is important for inhibiting structural damage within the gut. It was concluded that the reduced expression of *Muc2* and the increased expression of pro-inflammatory chemokines and cytokines may be associated with the increased abundance of the *A. muciniphilia* species in the gut. These findings raised concerns about the long-term health effects of CBD administration in terms of gastrointestinal health and suggested that further studies are needed [110].

Several of the studies described above concluded that CBD produces beneficial alterations of the gut microbiome, which subsequently leads to other positive physiological changes in several disease models. Yet, there is also controversy regarding the effect of CBD on the gut microbiome and potential therapeutic effects of CBD. For example, the study by Dopkins et al. concluded that CBD reduced neuroinflammation and intestinal inflammation, but CBD did not produce significant changes to the gut microbiome [109]. Therefore, further studies are needed to better understand the mechanism of action of the pharmacological effects of CBD on the microbiome, and how these effects alter other processes such as inflammation.

**Table 4:** Effect of CBD on the gut microbiome and its impact on diseases.

Disease	Model	CBD Doses and Routes of Administration	CBD Effect	Therapeutic Relevance	References
Inflammatory bowel disease	DSS induced in mice	Oral gavage (0.3–30 mg/kg)	CBD was administered either independently or with fish oil, ↑ *Akkermansia muciniphila* and *Parabacteroides goldsteinii*	Combination of fish oil and CBD may have therapeutic potential in the treatment of IBD	[90]
Non-alcoholic fatty liver disease	Mice induced with NAFLD	Oral gavage (2.39 mg/kg)	Improved glucose tolerance, ↓ inflammatory response markers such as TNF-alpha, and iNOS ↓ *Deferribacteres* levels and ↑ *Firmicutes* levels, which ↓ the *Bacteroidetes/Firmicutes* ratio ↑ *Clostridia*, *Ruminococcaceae* and *Bilophila* ↓ *Mucispirillum*	CBD may have a protective effect against NAFLD	[103]
Postmenopausal disorders	Ovariectomized mice	Oral gavage (25 mg/kg)	↑ relative abundance of fecal *Lactobacillus* species changes in intestine/femur gene expression that indicate ↓ bone resorption and ↓ inflammation Improved whole body bone mineral density	CBD may have usage to alleviate chronic symptoms of postmenopausal disorders	[104]
Epilepsy	Epileptic seizures were induced in rats using lithium chloride and pilocarpine	Oral gavage (20 mg/kg or 100 mg/kg)	Relieve dysbiosis of gut microbiome and ↓ metabolites involved in metabolic disorders ↑ in abundance of *Helicobacteraceae* and *Prevotellaceae UCG-100* ↓ the expression of pro-inflammatory markers such as IL-1β, IL-6, and TNF-α	CBD may be an effective treatment for microbiome dysbiosis associated with epilepsy	[108]
Experimental autoimmune encephalomyelitis (EAE)	EAE was induced in mice using myelin oligodendrocyte glycoprotein, Freund’s adjuvant, and dead Mycobacterium tuberculosis	Oral gavage (20 mg/kg)	↓ CXCL9, CXCL10, and IL-1β ↓ neuroinflammation and intestinal inflammation No effect on microbiome composition and SCFA production	CBD may be an effective treatment for inflammation in EAE, but had no effect on microbiome composition	[109]
Long-term effects of CBD usage	Mice fed with CBD-rich cannabis extract	Oral gavage (61.5 mg/kg)	↑ in abundance of *Akkermansia muciniphila*, which was associated with ↑ expression of pro-inflammatory cytokines and chemokines and ↓ expression of *Muc2*	Study raised concern about side effects of long-term CBD usage	[110]

Symbols used: ↑ increase; ↓ decrease/reduce; IBD, inflammatory bowel disease; NAFLD, non-alcoholic fatty liver disease; DSS, dextran sulfate sodium; iNOS, inducible nitric oxide synthase; IL-6, interleukin 6; IL-1β, interleukin 1 beta; TNF-α, tumor necrosis factor alpha; CBD, cannabidiol; EAE, experimental autoimmune encephalomyelitis; SCFA, short-chain fatty acid; CXCL, C-X-C motif chemokine ligand.

## 8. Future Directions and Conclusions

### 8.1. Mechanisms of Action for the Effects of CBD in Treating IBD and Diseases beyond the Gut

Many articles cited in this review studied the involvement of CBD molecular targets in the effects of CBD on intestinal epithelial cell permeability in vitro, using differentiated caco-2 cells. However, few studies have investigated whether these molecular targets for CBD expressed in the GI tract are involved in the potential therapeutic effects of CBD in vivo.

Previously two studies by Galiazzo et al. investigated the location of cannabinoid receptors within the ileum of healthy horses and dogs [111,112]. These studies showed that CBD molecular targets CB1, CB2, GPR55, and 5-HT1a had a wide distribution within the ileum [111,112]. However, while these studies were able to localize the CBD targets, there are no reports on their involvement in the mechanisms of action of CBD in vivo, e.g., in animal models of IBD. To improve our understanding of the role and function of CBD molecular targets in the gastrointestinal system, more mechanistic studies are necessary. Further in vivo studies using intestinal tissue-specific receptor knockout mice will help to elucidate the molecular mechanisms involved in the action of CBD within the GI system.

### 8.2. More Clinical Studies on the Effects of CBD for IBD and other Diseases outside of GI System

In 2018, the U.S. Food and Drug Administration (FDA) approved CBD (Epidiolex) for the treatment of Lennox–Gastaut syndrome and Dravet syndrome [113]. There is now keen interest in CBD as a potential therapy for a variety of diseases. Currently, there is a need for more human clinical trials to evaluate the potential effects of CBD on IBD. The number of human clinical trials on this topic is limited and the results have been inconsistent regarding efficacy of CBD. In the future, well-constructed, double-blind, and placebo-controlled trials using more patients from various ethnic groups and different doses and formulations of CBD are needed.

Previous studies have demonstrated that CBD is beneficial for diseases beyond the GI system. For example, through its modulatory actions on the gut microbiome, CBD may exert its well-known therapeutic effects on seizure disorders. In the future, it would be of great interest to conduct more clinical trials of CBD on other disorders and to study if and how the effects of CBD on gut microbiome play an important role in the potential therapeutic effects of CBD for diseases outside the GI tract.

### 8.3. Conclusions

CBD has exhibited modulatory effects on both the intestinal barrier permeability and the gut microbiome. In addition, CBD has displayed therapeutic potential for the treatment of GI disorders such as IBD. Furthermore, CBD may produce therapeutic effects on diseases outside the GI system by regulating gut–liver, gut–bone, and gut–brain axes.

In the future, it is important to elucidate the molecular and cellular mechanisms of actions of CBD in the gut. It is also crucial to perform more well-designed clinical trials to better understand the full therapeutic potential of CBD for disorders of the GI system and beyond.

## Figures and Tables

**Table 2:** Effect of CBD on inflammatory-disease animal models.

Model	CBD Doses and Routes of Administration	CBD Effect	Therapeutic Relevance	Reference
DNBS-induced colitis in mice	Intracolonic administration (1–10 mg/kg for gross evaluations and 5 mg/kg for histologic and expression measurements)	↓ of colon weight/length ratio ↓ swelling and ↑ gland regeneration ↓ inflammatory cytokines and iNOS ↓ production of ROS and lipid peroxidation	CBD is effective in treating DNBS-induced colitis	[87]
LPS-induced colitis in mice	Intraperitoneal injection (10 mg/kg)	↓ expression of S100B ↓ enteric glial cell activation and proliferation ↓ activation and prevalence of mast cells and macrophages within the intestine ↓ expression of TNF-α and cleaved caspase-3	CBD is effective in treating LPS-induced colitis	[88]
TNBS- and DSS-induced colitis in wild-type and CB2 knockout mice	Oral gavage (10 mg/kg)	Δ^9^-THC prevented colitis in wild-type but not in CB2 knockout mice CBD alone or in combination with Δ^9^-THC was not effective	CBD is not effective in treating TNBS- and DSS-induced colitis	[89]
DSS-induced colitis in mice	Oral gavage (0.3–30 mg/kg)	Neither fish oil nor CBD alone was effective for colitis Combination of fish oil and CBD ↓ colon inflammation and ↓ the colitis-associated increase in intestinal permeability	Combination of fish oil and CBD is effective in treating DSS-induced colitis	[90]

Symbols used: ↑ increase; ↓ decrease/reduce; IBD, inflammatory bowel disease; iNOS, inducible nitric oxide synthase; DNBS, dinitrobenzene sulfonic acid; ROS, reactive oxygen species; TNF-α, tumor necrosis factor alpha; TNBS, 2,4,6-trinitrobenzenesulphonic acid; DSS, dextran sulfate sodium; Δ^9^-THC, tetrahydrocannabinol; LPS, lipopolysaccharide.

**Table 3:** Human clinical studies which investigated the effect of CBD on inflammatory bowel disease.

Disease	Subjects	CBD Treatment	CBD Effect	Therapeutic Relevance	References
Crohn’s disease	19 patients with moderately active Crohn’s disease Mean age of 39 years	10 mg oral CBD twice daily for eight weeks	CBD had no significant effect on Crohn’s disease activity index No significant effects on liver or kidney function tests, hemoglobin levels, or albumin levels	CBD is well-tolerated at daily dose of 10 mg twice daily but did not exhibit efficacy for Crohn’s disease Future clinical studies should test higher doses of CBD	[91]
Ulcerative colitis	39 patients with mild to moderate ulcerative colitis Mean age of 44 years	Oral CBD-rich botanical extract for 10 weeks	CBD group participants exhibited lower severity scores, endoscopic subscores, and concentrations of circulating cytokines	CBD has therapeutic potential for ulcerative colitis Future research will focus on dosing to improve tolerability of the treatment in patients	[92]
